# Supplementary material for: Tunable directional photon scattering from a pair of superconducting qubits
Source: Nat Commun. 2023 May 24;14:2998. doi: 10.1038/s41467-023-38761-6 (PMC10209079; doi:10.1038/s41467-023-38761-6)
Supplement: Supplementary file 1 — Supplementary Information [file 41467_2023_38761_MOESM1_ESM.pdf]

Supplementary Information for: Tunable directional photon scattering from a pair of superconducting qubits

Elena S. Redchenko,[1,*] Alexander V. Poshakinskiy,[2] Riya Sett,[1]
Martin Žemlička,[1] Alexander N. Poddubny,[3] and Johannes M. Fink[1,†]

[1] *Institute of Science and Technology Austria, 3400 Klosterneuburg, Austria*
[2] *Ioffe Institute, St. Petersburg 194021, Russia*
[3] *Weizmann Institute of Science, Rehovot 7610001, Israel*

(Dated: April 28, 2023)

SUPPLEMENTARY METHODS

Supplementary Methods I: Modulation amplitude calibration. The measured frequency dependence of the modulation amplitude $A_m(\Omega)$ is shown in Fig. S1a. It is caused by the various low pass filter stages we use to suppress external flux noise on the bias input line. To calibrate the modulation amplitude A_m we measure the transmission spectrum $|t_0|^2$ of both modulated qubits at the amplitudes A_V from 0 to 0.1 $V_{\rm pp}$ and for different modulation frequencies Ω and fit it to Eq. 1. For each value of Ω, we fit the extracted A_m as a linear function of A_V as shown in Fig. S1b. After repeating this procedure for each qubit separately where one is tuned to $\omega_0/(2\pi) = 6.129\,\text{GHz}$ and the other is far ($> 2\,\text{GHz}$) detuned, we can now calculate the required A_V to result in the desired A_m for all Ω.

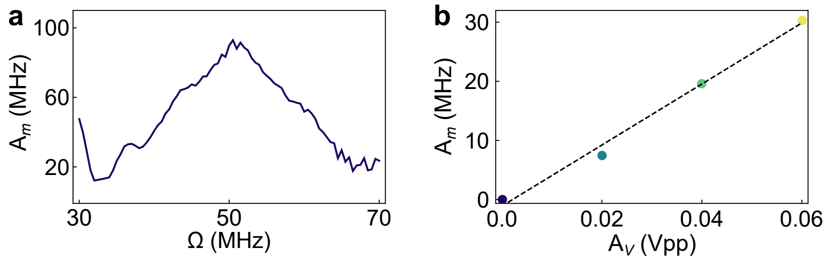

Supplementary Figure S1. **Modulation amplitude calibration. a,** Measured dependence of the modulation amplitude A_m on the modulation frequency Ω at the fixed AWG voltage amplitude $A_V = 0.2\,V_{\rm pp}$ obtained from a fit to Eq. 1. **b,** Measured dependence of A_m on the applied AWG amplitude A_V at the fixed modulation frequency $\Omega/(2\pi) = 20$ MHz.

Supplementary Methods II: Parameter optimization. Here we study optimal modulation parameters and tradeoffs between insertion loss and directivity with and without the presence of dephasing. For this we calculate forward and backward inelastic coherent scattering at $\alpha/\pi = 0$ phase difference between modulation tones for different modulation frequencies Ω and amplitudes A_m by numerically solving Eq. 7 in Methods. Figure S2 shows the predicted transmission power $P_\rightarrow$ (considering only the Stokes scattering) and directivity D for the experimentally extracted device parameters (panels a and c) as well as in the absence of pure dephasing when $\Gamma_2 = \Gamma_1/2$ (panels b and d). Generally speaking the highest levels of directivity are obtained for modulation parameters that show a lot of scattering to other frequency components and thus a higher insertion loss $1 - P_\rightarrow$. While insertion losses do not significantly change in the presence of dephasing, the directivity is actually improved, c.f. panels c and d.

The modulation parameters used in Fig. 3 of the main text ($\Omega/(2\pi) = 20\,\text{MHz}$, $A_m/(2\pi) = 20\,\text{MHz}$) are indicated with white crosses in Fig. S2a,c. At this point the transmission of 0.07 ($D = 0.45$) is close to the maximum possible of 0.08 with the experimental qubit properties. The modulation parameters used in Fig. 4 of the main text ($\Omega/(2\pi) = 20\,\text{MHz}$, $A_m/(2\pi) = 30\,\text{MHz}$) are indicated with black crosses. At this point the insertion losses are a bit higher ($P_\rightarrow = 0.05$) but so is the predicted directivity of 0.49, as also shown in Fig. 4 of the main text. For comparison, in the case of zero dephasing (shown in panels b and d) the highest transmission is 0.22 with a directivity of $D = 0.49$ achieved at a modulation frequency $\Omega \approx \Gamma_1/2$ and amplitude $A_m \approx 3\Gamma_1/2$, as indicated with the white cross.

It is important to clarify that what we refer to as insertion loss is mostly due to scattering to other frequency components. Such scattering can be suppressed using a structured waveguide forming a bandpass and has been

* elena.redchenko@ist.ac.at
† jfink@ist.ac.at

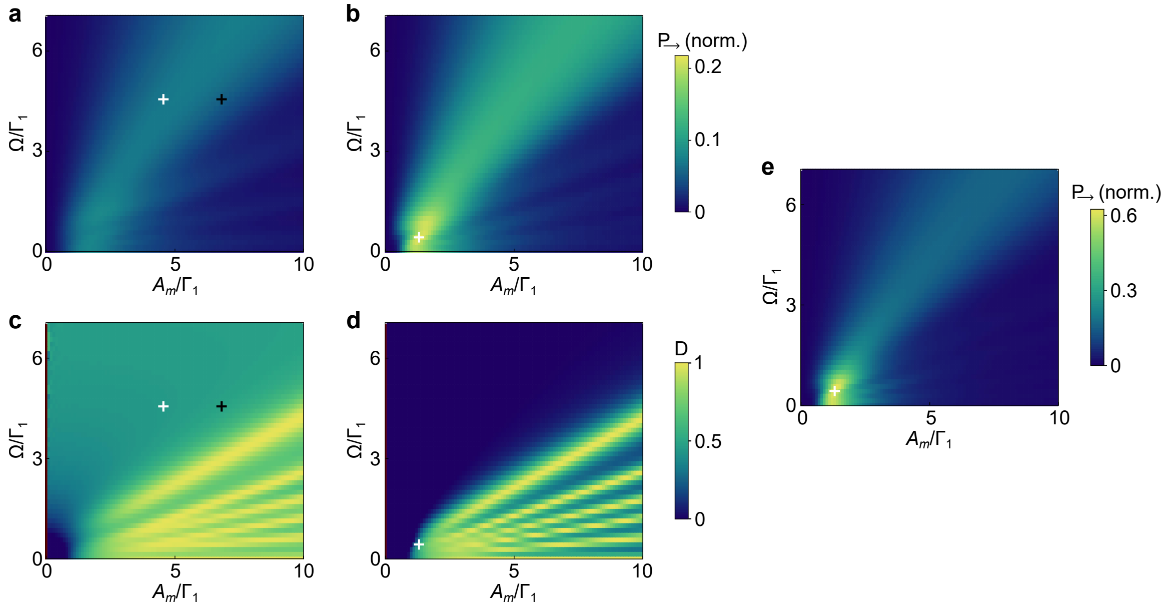

Supplementary Figure S2. **Modulation parameter dependence at** $\alpha = 0$**.** Numerically predicted insertion losses for the coherent inelastic scattering of the Stokes component as a function of modulation amplitude A_m and modulation frequency Ω for the experimentally extracted device parameters **a** and in the absence of pure qubit dephasing **b**. Corresponding, numerically calculated directivity as a function of A_m and Ω for the experimental parameters **c** and without pure dephasing **d**. White (black) crosses in **a** and **c** correspond to the modulation settings used in Fig. 3 (Fig. 4) of the main text. **e**, same as panel **b** but normalized to the remaining banpass filtered elastic and Stokes modes (forward and backward). White crosses in **b,d,e** correspond to the modulation parameters that minimize the insertion loss into the Stokes mode.

demonstrated in Ref. [1]. Such a waveguide would act as a filter around the qubits central ω_0 and Stokes $\omega_0 - \Omega$ frequencies. A rigorous estimation of the insertion loss suppression would require a modification to our theoretical approach. Namely, the photon Green's function term $\propto -\mathrm{i}e^{\mathrm{i}\varphi|j-k|}$ in Eq. (5) in Methods would have to be replaced by a Green's function of a photon in a structured waveguide. The modified Green's function would also depend on the photon frequency, which means including the non-Markovian effects. While such consideration is straightforward, it requires a separate detailed study that is out of the scope of the current work. Instead, we use here a simpler approach to provide the lower bound for the insertion loss. By integrating the total power in unwanted sidebands, i.e. all but the elastic and Stokes frequencies in both reflection and transmission, we estimate a resulting reduction of insertion loss by a factor of 2.8 in the absence of dephasing. This would correspond to a remaining insertion loss of -2.1 dB for the coherently scattered light into the Stokes mode. About one-fourth of the remaining loss is caused by elastic scattering. We note that while in our simplified approach we assumed that the ratio between scattering into the allowed inelastic and elastic channels was not modified by the structured waveguide, the emission process might also be modified by the bandpass. Nonetheless, we see in our optimization that the remaining insertion loss is mainly due to limited directivity. A better trade-off between directivity and insertion loss might be possible to find for different values of α, e.g. in the experimental data (Fig. 4e) the highest level of directivity is found at $\alpha = 0.1\,\pi$.

Supplementary Methods III: Device bandwidth. The bandwidth of the elastically scattered light interacting with a modulated qubit can readily be calculated from Eq. 1 in the main text, as shown in Fig. 2a. In contrast, the transmission spectrum of the inelastic scattered light for one or two modulated qubits needs to be calculated numerically. Figure S3 shows the measured (dots) and calculated (solid line) reflected inelastically scattered light at $\alpha = -\pi$ where it is maximal. The observed 4 resonances at probe frequencies ω_0, $\omega_0 \pm \Omega$, and $\omega_0 - 2\Omega$ all have a bandwidth of approximately $\approx \Gamma_{2,\text{qubit }1} + \Gamma_{2,\text{qubit }2}$ as indicated by the Lorentzian fit (dashed line). This is a good approximation for device bandwidth in the sideband resolved limit. In the experimental situation shown in Fig. S3, where $A_m/(2\pi) = 30\,\text{MHz}$ and $\Omega/(2\pi) = 20\,\text{MHz}$ (as in Fig. 4 of the main text) the full measured device bandwidth at half maximum value is $\approx 25\,\text{MHz}$ as indicated by black arrows.

Supplementary Methods IV: Power dependence of directionality. In this section, we discuss the dependence of the directionality on the incident wave power. In order to calculate the scattering we use the master equation [2]

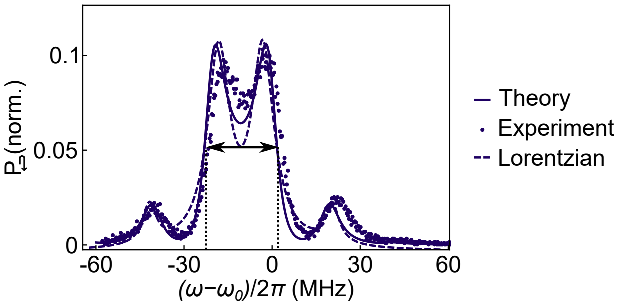

Supplementary Figure S3. **Inelastic reflection spectrum.** Measured coherent inelastic backward scattering at $\alpha = -\pi$ as a function of $\omega - \omega_0$ (points), theory (solid line), and Lorentzian fit (dashed line) for $A_m/(2\pi) = 30\,\mathrm{MHz}$ and $\Omega/(2\pi) = 20\,\mathrm{MHz}$. The device bandwidth (black arrows) is indicated at half the maximum value of the central component.

for the density matrix ρ:

$$\dot{\rho} = -i[H_1, \rho] + \sum_{j,k=1}^{N} \left(\frac{\Gamma_1}{2} \cos[\varphi(j-k) + \Gamma_2 \delta_{j,k} \right) \left[2\sigma_j \rho \sigma_k^\dagger - \{\sigma_k^\dagger \sigma_j, \rho\} \right] \tag{S1}$$

with the Hamiltonian

$$\begin{aligned} H_1 &= \sum_{j=1}^{N} [\omega_0 + A_m(t) \cos(\Omega t + \alpha_j)] \sigma_j^\dagger \sigma_j + \tfrac{\Gamma_1}{2} \sum_{j,k=1}^{N} \sigma_j^\dagger \sigma_k \sin(\varphi |j-k|) \\ &+ \sum_{j=1}^{N} \tfrac{\Omega_R}{2} (\mathrm{e}^{-\mathrm{i}\varphi j - \mathrm{i}(\omega-\omega_0)t} \sigma_j^\dagger + \mathrm{H.c.}) \,. \end{aligned} \tag{S2}$$

The coherent reflection and transmission coefficients that describe the backward (forward) scattering process with the frequency change $\omega \to \omega + n\Omega$ are then found as the Fourier transforms

$$r_n = \frac{2}{\Omega_R} \lim_{T\to\infty} \frac{1}{T} \int_0^T \mathrm{d}t\, \mathrm{e}^{-\mathrm{i}(\omega+n\Omega)t} \sum_{j=1}^{N} [\mathrm{Tr}\, \rho(t) \sigma_j^\dagger] \mathrm{e}^{\mathrm{i}\varphi j} \,, \tag{S3}$$

$$t_n = \delta_{n,0} + \frac{2}{\Omega_R} \lim_{T\to\infty} \frac{1}{T} \int_0^T \mathrm{d}t\, \mathrm{e}^{-\mathrm{i}(\omega+n\Omega)t} \sum_{j=1}^{N} [\mathrm{Tr}\, \rho(t) \sigma_j^\dagger] \mathrm{e}^{-\mathrm{i}\varphi j} \,. \tag{S4}$$

The calculated forward and backward scattering spectra $P_{\rightarrow} \equiv |t_n|^2$ and $P_{\hookleftarrow} \equiv |r_n|^2$ are shown in Fig. S4. In the limit of vanishing driving power this calculation yields the same results as the one presented in the main text. Namely, the $\alpha = \pi$ phase difference between the modulation tones of first and second qubits corresponds to strong inelastic backscattering, see Fig. S4d. Increase of the Rabi frequency leads to a gradual suppression of the scattering directionality that persists up to $\Omega_\mathrm{R} \approx 3\Gamma_1$. This can be interpreted as a result of the saturation of the qubit transition induced by a strong driving [3].

Supplementary Methods V: Isolator properties. According to our theory, isolator behavior is expected at the relative phases $\alpha/\pi \approx \pm 0.4$, where the reflection in both directions is strongly suppressed and where there is an asymmetry in transmission in opposite directions resembling the S-matrix of an ideal isolator

$$S_\mathrm{isolator} = \begin{pmatrix} 0 & 0 \\ 1 & 0 \end{pmatrix} . \tag{S5}$$

For the elastic measurements at a modulation frequency $\Omega/(2\pi) = 20\,\mathrm{MHz}$ and modulation amplitude $A_m/(2\pi) = 30\,\mathrm{MHz}$, we find an isolation of $10\log_{10}(S_{21}/S_{12}) \approx 1.4\,\mathrm{dB}$ and an insertion loss of $10\log_{10} S_{21} \approx 2\,\mathrm{dB}$ at $\alpha/\pi \approx 0.4$ as shown in Fig. S5a which is in qualitative agreement with the theoretically predicted isolator scattering matrix shown in Fig. S5b.

We also observe the reversed isolation process for the inelastically scattered light as shown in Fig. S5c(d). For

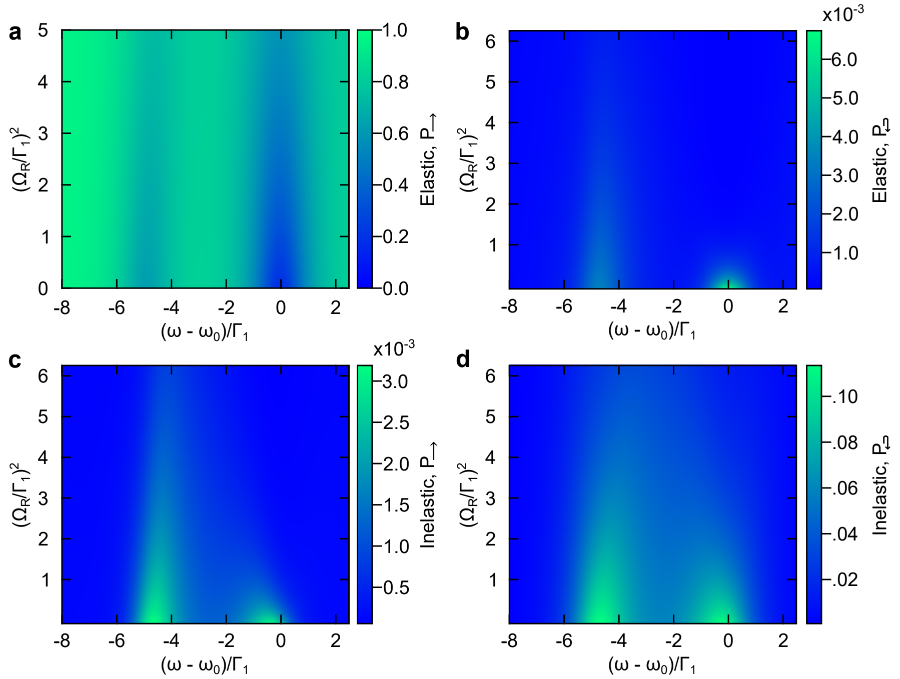

Supplementary Figure S4. **Dynamic range of directional scattering.** Calculated coherent elastic (**a, b**) and inelastic (**c, d**) scattering spectra as a function of normalized drive frequency $(\omega - \omega_0)/\Gamma_1$ and drive power $(\Omega_R/\Gamma_1)^2$ theoretically predicted for reflection (**a, c**) and transmission (**b, d**). The calculation has been performed for the modulation phase difference $\alpha_2 - \alpha_1 = \pi$ and $\Omega = A_m = 5\Gamma_1$.

the same modulation parameters $\Omega/(2\pi) = 20\,\text{MHz}$ and $A_m/(2\pi) = 30\,\text{MHz}$ and the relative phase $\alpha/\pi \approx 0.4$, we measure an isolation of $10\log_{10}(S_{12}/S_{21}) \approx 2.7\,\text{dB}$ and an insertion loss of $10\log_{10} S_{12} \approx 11.1\,\text{dB}$.

Such a non-reciprocity is based on the travelling-wave modulation similar to the electrically induced non-reciprocity in the optical domain [4]. In our system, the isolation strength can be improved by optimizing the modulation amplitude. Moreover, adding more qubits to the device while keeping the effective distance between nearest neighbors at $\lambda/4$ and the relative phase $\alpha/\pi = 0.5$ between modulation tones would enable the creation of a topological isolator for both elastically and inelastically scattered light [5–7].

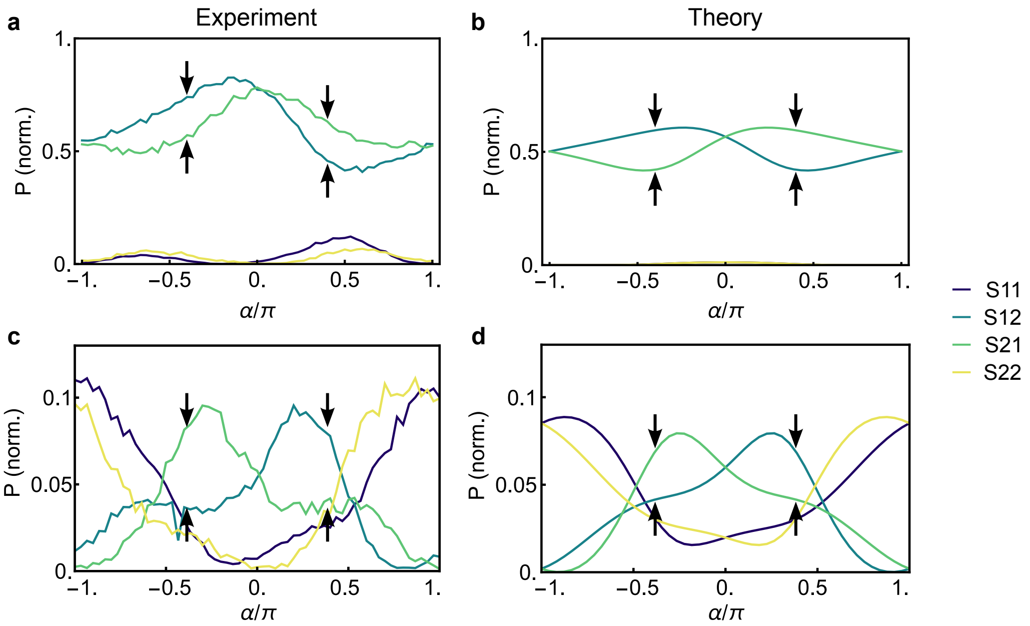

Supplementary Figure S5. **Isolator scattering matrix. a.** Measured coherent elastic scattering power normalized to the background transmission coefficient shown as a function of the relative phase between modulation tones α where $\Omega/(2\pi) = 20\,\mathrm{MHz}$ and $A_m/(2\pi) = 30\,\mathrm{MHz}$. **b.** Theoretically predicted elastic scattering. **c.** Measured coherent inelastic scattering power of the Stokes component normalized to theory shown as a function of the relative phase between modulation tones α where $\Omega/(2\pi) = 20\,\mathrm{MHz}$ and $A_m/(2\pi) = 30\,\mathrm{MHz}$. **d.** Theoretically predicted inelastic scattering.

REFERENCES

[1] V. S. Ferreira, J. Banker, A. Sipahigil, M. H. Matheny, A. J. Keller, E. Kim, M. Mirhosseini, and O. Painter, Collapse and revival of an artificial atom coupled to a structured photonic reservoir, Physical Review X **11**, 041043 (2021).

[2] K. Lalumière, B. C. Sanders, A. F. van Loo, A. Fedorov, A. Wallraff, and A. Blais, Input-output theory for waveguide QED with an ensemble of inhomogeneous atoms, Phys. Rev. A **88**, 043806 (2013).

[3] O. Astafiev, A. M. Zagoskin, A. Abdumalikov, Y. A. Pashkin, T. Yamamoto, K. Inomata, Y. Nakamura, and J. S. Tsai, Resonance fluorescence of a single artificial atom, Science **327**, 840 (2010).

[4] H. Lira, Z. Yu, S. Fan, and M. Lipson, Electrically driven nonreciprocity induced by interband photonic transition on a silicon chip, Physical review letters **109**, 033901 (2012).

[5] A. Celi, P. Massignan, J. Ruseckas, N. Goldman, I. B. Spielman, G. Juzeliūnas, and M. Lewenstein, Synthetic gauge fields in synthetic dimensions, Phys. Rev. Lett. **112**, 043001 (2014).

[6] L. Yuan, Y. Shi, and S. Fan, Photonic gauge potential in a system with a synthetic frequency dimension, Opt. Lett. **41**, 741 (2016).

[7] A. N. Poddubny and L. E. Golub, Ratchet effect in frequency-modulated waveguide-coupled emitter arrays, Phys. Rev. B **104**, 205309 (2021).
